# Challenges and Opportunities in Evaluating Novel Drug-Radiotherapy Combinations

**DOI:** 10.7759/cureus.103207

**Published:** 2026-02-08

**Authors:** Shelby Lane, Charles B Simone, Jessica Schuster, Jason Efstathiou, Steven Eric Finkelstein, Vatsal Patel, Yun Li, Susannah G Ellsworth, Mitchell Oscar Finkelstein, Thomas Kim, Lawrence R Kleinberg, Michael Tomblyn

**Affiliations:** 1 Radiation Oncology, Baptist Memorial Hospital, Memphis, USA; 2 Radiation Oncology, New York Proton Center, New York, USA; 3 Radiation Oncology, University of Wisconsin, Madison, USA; 4 Radiation Oncology, Massachusetts General Hospital, Boston, USA; 5 Associated Medical Professionals of New York, US Urology Partners, Syracuse, USA; 6 Radiation Oncology, I Am Still Alive, LLC, Phoenix, USA; 7 Radiation Oncology, City of Hope Comprehensive Cancer Center, Duarte, USA; 8 Radiation Oncology, University of Pittsburgh Medical Center, Pittsburgh, USA; 9 Barrett, The Honors College, Arizona State University, Tempe, USA; 10 Radiation Oncology, Rush University Medical Center, Chicago, USA; 11 Radiation Oncology, Johns Hopkins University School of Medicine, Baltimore, USA

**Keywords:** clinical trials, immunotherapy, oncology, radiotherapy, research

## Abstract

Given the recent explosion in the number and diversity of novel targeted systemic therapies, many future advances in cancer treatment may result from improving the therapeutic ratio of radiotherapy (RT) by exploiting combinations of RT and novel targeted systemic therapies. Despite the abundance of preclinical models suggesting that new agents can improve the efficacy of RT, early-phase clinical trials have not demonstrated expected synergies or even additive efficacy between RT and these agents, particularly in trials of concurrent immunotherapy/RT. This underscores the ongoing need to develop well-formulated and well-executed early-phase clinical trials in this space that are based on sound science and are well-positioned to discern the optimal ways to combine these treatment modalities. What are the barriers to the completion of early-phase clinical trials combining RT and drugs?

## Editorial

The recent review by Zumsteg et al. [1], featured in The Lancet Oncology, meticulously addresses the key critical challenges and actionable strategies in designing early-phase clinical trials for drug-radiotherapy (RT) combinations. Their analysis stands as an insightful beacon for continued innovation and advocates for interdisciplinary approaches and evidence-based solutions to advance oncology therapeutic paradigms.

Zumsteg et al. begin by elucidating the unique challenges of investigating these combinatorial strategies in early-phase trials. The lack of reliable preclinical models for concurrent therapies represents a crucial weakness of our current system, compounded further by the rise in importance of immunotherapeutic agents as the immune system of healthy young mice often is poorly representative of the ill oncology patient with numerous confounding comorbidities seen in clinical practice. Additionally, the prevailing models of developing systemic anticancer therapies generally are assessed in patients who have already failed multiple lines of therapy, are in poor condition, and/or have little likelihood of surviving long enough to assess for long-term complications of the novel agent(s). The current models of assessment of dose-limiting toxicities (DLTs) in early-phase studies are not well-positioned to assess for acute and subacute complications of concurrent treatment approaches that often take weeks to months to complete. Further challenges of heterogeneity of effects based upon anatomic location and the paucity of regulatory guidance for concurrent approaches present additional complications to the investigator. Finally, the complexity and variability of the antitumor immune response and the difficulty of obtaining serial tumor samples following RT pose significant challenges for biomarker development [2]. However, identifying reliable peripheral markers for the induction of antitumor immune responses is a critical step in developing studies to rapidly and accurately screen novel RT/immuno-oncology (IO) combinations.

Next, the authors deliver a comprehensive analysis of the myriad design options for early-stage clinical trials, reflecting on both the advantages and disadvantages of each approach. The statistical designs most familiar to the oncologist suffer from weaknesses of slow pace, inflexibility, and the elevated risk of exposing more subjects to toxicity. Often, the sample sizes utilized are too small to adequately assess for the maximum tolerable dose (MTD) in concurrent therapies. Discerning the relatedness of documented toxicities between the interventions used, already challenging in multidrug trials, is more confounding when combining RT with systemic therapy. The authors present more rigorous trial options, such as the time-to-event continuous reassessment method (CRM), biomarker-driven patient selection, and Bayesian designs, with which most oncologists are currently unfamiliar and that typically require more intensive statistical support.

Having set forth the unique challenges of designing these concurrent clinical trials, the authors offer a glimpse of opportunities that may lend themselves to novel investigations of drug-RT combinations. Unlike medical oncology's traditional focus on testing new treatments for patients who have exhausted other options, radiation oncology offers promising opportunities to address unmet needs across multiple clinical settings: definitive, adjuvant, neoadjuvant, and the evolving field of oligometastatic disease. The authors highlight the unique challenges with the inclusion of RT, with some combinations being perfectly reasonable in one anatomic location but quite intolerable in another due to the heterogeneity of normal tissue tolerances of different sites.

The authors conclude with an optimistic view of opportunities for investigating novel drug-RT combinations, expressing confidence that none of the aforementioned challenges are insurmountable. While the extensive statistical, biobanking, and institutional infrastructure of national clinical trial cooperative groups theoretically positions these organizations to address the challenges of investigating novel drug-RT combinations, these organizations have also been hampered by recent uncertainties regarding federal support for cancer research, delays in clinical trial activation and reporting, restrictive clinical trial eligibility criteria [3], and difficulties in accessing biospecimens for secondary analysis [4]. It is imperative to develop new clinical trial and collaborative mechanisms to advance the pace at which promising therapies are brought to the clinic.

Only small, single-digit percentages of eligible cancer patients elect to participate in clinical trials [5]. Evaluating and understanding the unique challenges of clinical trial design and recruitment are essential, particularly within the community setting where the vast majority of patients receive their care.

**Figure 1:**
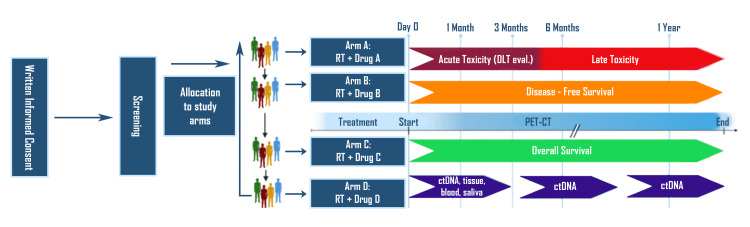
Hypothetical drug-RT platform studies. Clinical trial patients, potentially selected based on clinical factors and radiosensitivity index biomarkers, are enrolled initially to a specific drug-RT combination (drug A-RT). When a prespecified number of patients have been enrolled, subsequent patients are enrolled on another drug-RT combination (drug B-RT), while drug A-RT patients progress through the dose-limiting toxicity window. Drug B-RT enrolls a prespecified number of patients; subsequent patients are enrolled to receive drug C-RT, followed by drug D-RT, until eventually returning to enroll in drug A-RT. RT: radiotherapy; PET-CT: positron emission tomography-computed tomography; ctDNA: circulating tumor DNA

Figure 1 represents hypothetical drug-RT platform studies as initially proposed by Zumsteg et al. [1].

It is well-known that disparities exist in the distribution of oncologic care in the US rural-urban continuum. This harsh reality has only worsened in the past few years, and cancer health outcomes in the future will reflect that. This inequity is particularly vast for radiation oncology given the multi-disciplinary nature of the specialty involving several teams within the specialty itself, including physician(s), dosimetry, physics, radiation therapy team, nursing/medical assistant(s), a highly complicated billing, and arguably the most complicated billing of any medical specialty. It is more important now than ever to strategically recruit dedicated and compassionate medical professionals who may not be part of big groups or come from academia but may have substantial contributions to make towards science and humanity. It is also imperative that we ensure that all populations in the United States and around the world are represented in these clinical trials, since what works for one population may not be generalized to all populations. While such challenges already exist for clinical trials, they will be exacerbated further when considering the lack of support staff for research as well as access to drugs and the full extent of radiation oncology resources to support standard of care practices. As a society, it is our responsibility to ensure that elite care is not reserved only for the elites but made available to the most vulnerable sections of societies, whether in the United States or elsewhere. This also means the more privileged have a responsibility to reduce barriers historically placed for doctors who do not belong in the elite cadre of doctors. To that end, academic scholarship for the underprivileged doctors will translate into better outcomes for the underserved patients by meaningfully engaging doctors from community practices everywhere, with or without affiliations with cooperative groups or academia. Real clinical practice experience often leads to the best ideas for clinical trials, and it is important not to ignore the varied experiences of all doctors from all geographies and settings.

The Lancet Oncology article also represents a new avenue of opportunity for the American College of Radiation Oncology (ACRO). ACRO recently launched the Contemporary Updates: Radiotherapy Innovation & Evidence (CURiE) journal as the official publication platform for the College. Until recently, ACRO has not been involved in developing white papers in radiation oncology. The early-phase drug-RT combination white paper was led by NRG Oncology's Developmental Therapeutics and Radiation Therapy Subcommittee, who solicited interdisciplinary investigators from the American Society for Radiation Oncology (ASTRO), the American College of Radiology (ACR), the Sarah Cannon Research Institute, and ACRO. This was one of the first times ACRO has been involved in such a white paper with other societies, and for it to be performed for such an important and timely topic in such a high-impact journal as The Lancet Oncology provides a new voice for ACRO members. ACRO selected a member to participate in the development of the white paper (SEF), and then the completed article underwent review for endorsement by the ACRO Science and Education Committee (led by CBS and MBT). This paves the way for the potential for future positions and white papers to be generated representing ACRO's voice through the Science and Education Committee, which is crucial for the continued success and further growth of the College.
